# Patient and Clinician Perceptions of Factors Relevant to Ideal Specialty Consultations

**DOI:** 10.1001/jamanetworkopen.2022.8867

**Published:** 2022-04-25

**Authors:** Stephanie D. Roche, Anna C. Johansson, Jaclyn Giannakoulis, Michael N. Cocchi, Michael D. Howell, Bruce Landon, Jennifer P. Stevens

**Affiliations:** 1 Department of Health Care Quality, Beth Israel Deaconess Medical Center, Boston, Massachusetts; 2 Center for Health Care Delivery Science, Beth Israel Deaconess Medical Center, Boston, Massachusetts; 3 Department of Medicine, Beth Israel Deaconess Medical Center, Boston, Massachusetts; 4 Harvard Medical School, Boston, Massachusetts; 5 Patient and Family Advisory Council, Beth Israel Deaconess Medical Center, Boston, Massachusetts; 6 Department of Emergency Medicine, Beth Israel Deaconess Medical Center, Boston, Massachusetts; 7 Department of Anesthesia, Critical Care & Pain Medicine, Beth Israel Deaconess Medical Center, Boston, Massachusetts; 8 Google Health, Palo Alto, California; 9 Department of Health Care Policy, Harvard Medical School, Boston, Massachusetts; 10 Department of Pulmonary, Critical Care, and Sleep Medicine, Beth Israel Deaconess Medical Center, Boston, Massachusetts

## Abstract

**Importance:**

Inpatient subspecialty consultations, a common and expensive practice within inpatient medicine, do not always go well; however, little is known about the failure modes of consultation, thus making it difficult to identify interventions to improve consultation quality.

**Objective:**

To understand how stakeholders envision the ideal inpatient consultation and identify how and why consultations commonly fall short of this ideal.

**Design, Setting, and Participants:**

This qualitative study used in-depth, semistructured interviews collected from April to October 2017 and analyzed from January 2018 to February 2020 using conventional content analysis. The setting was a single academic medical center in Boston, Massachusetts. Participants were hospitalists and specialists who had requested or performed a consultation for a non–intensive care unit patient in the previous 4 months, patients who had received a consultation while hospitalized at the medical center in the previous 15 months, and family members of such patients.

**Main Outcomes and Measures:**

Consultation experiences reported by participants. Clinicians were asked about characteristics of the ideal consultation, positive and negative consultation experiences, costs and benefits, and suggested improvements. Patients and family members were asked about their consultation experience, changes in care, communication preferences, and suggested improvements.

**Results:**

The study included 38 participants: 17 specialists, 13 hospitalists, 4 patients, and 4 family members. More than half (21 of 38) of the participants were female. There were 11 key information exchanges identified that occur among the specialist team, primary team, and patient/family during an ideal consultation. These exchanges are time sensitive and primarily carried out through unwritten protocols. We also identified 6 defects (process failures) that commonly derail information exchanges (complete omission, exclusion of a key stakeholder, poor timing, incomplete or inaccurate information, and misinterpretation) and 5 contextual factors (roles and boundaries, professionalism, team hierarchy, availability, and operational know-how) that influence how information exchange unfolds, making some consultations more prone to defects.

**Conclusions and Relevance:**

Successful inpatient consultation requires a complicated, sequenced series of time-sensitive information exchanges that are highly vulnerable to failure. Maximizing the benefit of consultations will likely entail not only minimizing low-value consultations but also actively preventing defects, such as information inaccuracies and misinterpretation, that commonly derail the consultation process.

## Key Points

**Question:**

How do stakeholders envision the ideal inpatient consultation and in what ways do consultations commonly fall short of this ideal?

**Findings:**

In this qualitative study of 17 specialists, 13 hospitalists, 4 patients, and 4 family members, participants identified 11 key information exchanges that occur during an ideal consultation, as well as 6 common defects and 5 contextual factors that influence how these information exchanges transpire.

**Meaning:**

These findings suggest that successful inpatient consultation requires a complicated, sequenced series of time-sensitive information exchanges guided primarily by unwritten norms and highly vulnerable to failure.

## Teaser

This qualitative study examines how stakeholders envision the ideal inpatient specialty consultation and why consultations commonly fall short of this ideal.

## Introduction

Inpatient subspecialty consultation—engagement of a specialist for additional clinical expertise or specialty procedures—is a common and expensive practice within inpatient medicine. In one analysis of 3.1 million admissions of Medicare patients from 2009 to 2010, patients received 2.6 consultations per admission on average.^1^ A separate study of 736 000 admissions of Medicare patients found subspecialty consultative care accounted for $1.3 billion of Medicare spending in 2014.^2^ Given the ubiquity and expense of inpatient consultations, research has focused heavily on assessing variation in consultation use^1,3,4,5^ and association with patient-level outcomes.^6,7,8,9,10^

A third research area centers on assessing communication among consultation stakeholders^11,12,13,14,15,16,17^ and identifying factors that influence consultation “effectiveness.”^11,13^ Although useful for understanding consultation context, such studies do not provide complete insight into how consultations fail, making it difficult to identify interventions. We interviewed hospitalists, specialists, patients, and family members to understand how these stakeholders envisioned the ideal inpatient consultation and ways consultations commonly fall short of this ideal. We hypothesized that different stakeholder groups would vary in their characterization of the ideal consultation and identify multiple points of potential failure.

## Methods

This study was approved by the institutional review board of Beth Israel Deaconess Medical Center. All participants provided written informed consent. We followed the Consolidated Criteria for Reporting Qualitative Research (COREQ) reporting guideline.

### Setting and Eligibility Criteria

This study took place at a single academic medical center in Boston, Massachusetts. Study participants included English-speaking adults who were (1) hospitalists, (2) specialists, (3) patients, or (4) family members of patients. Our sampling frame for physicians included residents, fellows, and attendings who had requested or performed a consultation for a non–intensive care unit patient in the previous 4 months. Our sampling frames for patients and family members were, respectively, patients who had received a consultation while hospitalized at the medical center in the previous 15 months and family members of such patients.

### Sampling and Recruitment

This study used purposive sampling. A research assistant (RA) emailed the medical center’s hospitalist and medical residency program listservs describing the study and followed up with interested individuals. After each interview, we asked participants for suggestions as to whom else to interview. We invited members of the medical center’s Patient and Family Advisory Council to participate.^18,19,20^

### Instrument Development

Our semistructured interview guides (eAppendix 1 in the Supplement) asked clinicians about the ideal consultation, costs and benefits, positive and negative consultation experiences, and suggested improvements; and patients/family members were asked about their consultation experience, changes in care, communication preferences, and suggested improvements. We pilot-tested the guides with 2 to 4 individuals from each sampling frame and revised them for clarity.

### Data Collection

From April to October 2017, author S.D.R.—a female RA with master’s-level training in qualitative research—interviewed each participant once in a private conference room or via phone. S.D.R. had no prior relationship with any participants. Each interview was recorded, transcribed verbatim, and spot-checked for accuracy.

### Data Analysis

We analyzed data in NVivo 12 (QSR International) using conventional content analysis from January 2018 to February 2020.^21^ S.D.R. and A.C.J.—a coinvestigator with doctoral training in qualitative research—independently reviewed a sample of transcripts and developed a preliminary codebook, which they further refined with authors J.P.S. and J.G. The final codebook included 44 codes capturing information exchange processes and 5 codes capturing relevant contextual factors. All transcripts were coded by author S.D.R. and checked by author A.C.J., with disagreements resolved through group discussion. We performed second-cycle pattern coding^22,23^ to arrange the 44 process codes into discrete information exchanges (eTable 1 in the Supplement) and defects (process failures). eTable 2 in the Supplement includes additional details about our methods.

## Results

### Participants

We interviewed 38 individuals: 17 specialists (7 [41%] were female), 13 hospitalists (6 [46%] were female), 4 patients (all were female), and 4 family members (all were female). Approximately one-third of interviews (12 of 38) were conducted in-person, with the remainder conducted by phone. Median (IQR) interview duration was 29 (24-34) minutes.

Aside from one third-year primary team resident, all clinicians were attendings. Specialists included 5 physicians who specialized in surgery; 3 in cardiology; 1 in neurology; and 2 each in gastroenterology, infectious disease, hematology/oncology, and pulmonology.

### Theme 1: Information Exchanges

The first theme identified was that the ideal consultation features a series of information exchanges that were dictated by mostly unwritten protocols performed during distinct stages of the consultation process. Collectively, participants identified 11 primary information exchanges (IEs) that occur in an ideal consultation (Table 1; eAppendix 2 in the Supplement). These IEs featured clinical information (eg, patient’s medical history) and process information (eg, “consult request received”) that signal “whose court the ball is in” as the work passes between the primary and specialist teams (Figure). Many clinicians noted that the consultation process was largely guided by informal protocols. According to one hospitalist, “Most consultations are typically seen and staffed by an attending within 24 hours. At least that’s sort of an unwritten expectation” (Participant 12).

**Table 1.** Primary Information Exchanges That Occur in the Ideal Consultation

Consultation stage	Information exchange		Interactants		Information given/solicited by interactant	
	No.	Name	1	2	1	2
Identification of consultation need	IE 1	Initial question formation	Primary team	Primary team^a^	Consultation question Rationale for needing consultation	Confirmation: understand consultation question and rationale
	IE 2	Patient/family go-ahead	Primary team	Patient/family	Why primary team wants specialist input What the consultation entails Answers to any patient/family questions	Confirmation: understand & agree with consult Questions for primary team about proposed consult
ConsultationRequest	IE 3	Consultation request	Primary team	Specialist team	Who patient is Consultation question Other relevant patient information	Confirmation: received request & understand consultation question What other information, if any, needed to begin consult Expected timeframe for patient evaluation
	IE 4	Request processing	Specialist team^b^	Specialist team^c^	Consultation question Plan for conducting consult	Confirmation: agree with plan
	IE 5	Timeframe estimate	Primary team	Patient/family	Expected timeframe for patient evaluation	Confirmation: understand expected timeline
Patient evaluation	IE 6	Patient evaluation	Specialist team	Patient/family	Questions about patient (eg, medical history) Answers to primary team’s questions	Answers to specialist team’s questions Questions for specialist team
	IE 7	Patient evaluation follow-up	Specialist team	Primary team	Questions about patient Expected timeframe for final recommendations	Answers to specialist team’s questions
Recommendation formation, consensus building, and finalization	IE 8	Consensus among specialist teams	Specialist team	Specialist team	(If multiple specialist teams consulting) Preliminary recommendations (If applicable: trainee to attending) Preliminary recommendations	(If multiple specialist teams consulting) Feedback & consensus on recommendations (If applicable: attendee to trainee) Confirmation: agree with preliminary recommendations
	IE 9	Recommendation	Specialist team	Primary team	Recommendations (If applicable) Confirmation: attending specialist vetted recommendations Answers to primary team’s questions	Confirmation: received final recommendations Clarifying questions for specialist team
Recommendation implementation	IE 10	Communication of recommendations to patient/family	Primary/ specialist team	Patient/family	Updated care plan Answers to patient/family’s questions	Confirmation: understand & agree with updated care plan Questions for primary team or specialist team
	IE 11	Recommendation action	Primary team	Specialist team	(If recommendations not implemented) Reason why not implemented	Confirmation: understand & agree with non-implementation of recommendations

Abbreviation: IE, information exchange.

^a^ Primary team member responsible for inputting the consultation request.

^b^ Specialist team member responsible for receiving consultation request; commonly a trainee in academic medical centers.

^c^ Specialist team member who will staff the consultation or coconduct the consultation with a trainee.

**Figure.**
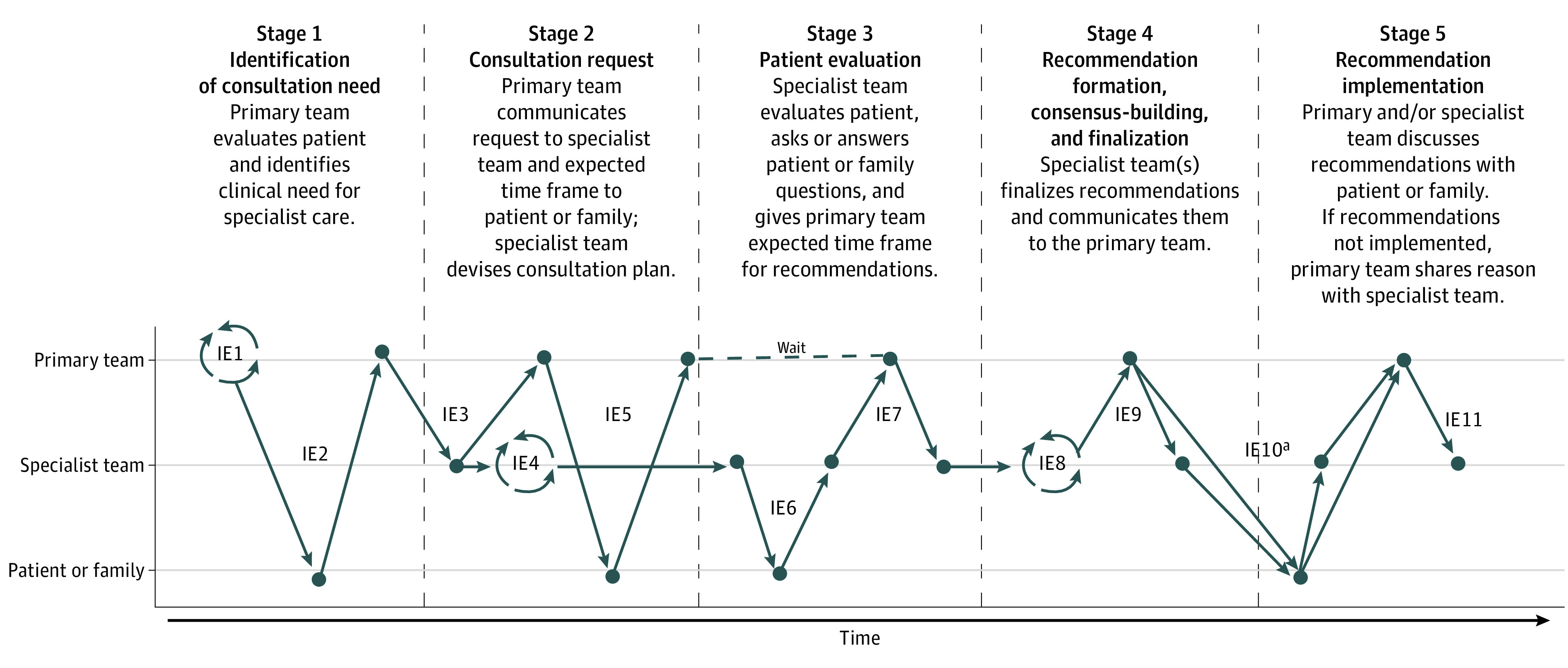
Eleven Primary Information Exchanges (IE) Among Interactants During an Ideal Consultation

Across teams and specialties, clinicians’ descriptions of the ideal consultation were similar in their key processes, division of labor, and flow, with nearly all dividing it into 5 main stages (Figure). Patients and families participated in the consultation process but in a more limited way, focusing on whether they were notified of the consultation in advance, their interaction with the specialist, and what ultimately resulted from the consultation.

### Theme 2: Defects (Process Failures)

The second theme identified was that in many consultations, information exchanges were derailed by 1 or more defects (process failures). Participants identified 6 defects (Table 2) that commonly derail the IEs in the Figure and lead to confusion, frustration, and/or care inefficiencies. Defects included an IE (1) being completely omitted or (2) occurring too early or late; (3) excluding 1 or more key stakeholders; (4) featuring incomplete or (5) inaccurate information; or (6) involving misinterpretation of otherwise complete, accurate information by 1 or more interactants. Participants described some IEs as more vulnerable to defects than others, and that problems often compound throughout the consultation, with a defect in one IE contributing to defects in later IEs. Noting the high prevalence of defects, some participants expressed surprise that consultations do not lead to serious patient harm more often.

**Table 2.** Six Common Defects of Information Exchanges Occurring During Inpatient Consultations

Defect domain-defect	Description	Example	Illustrative quotes
Process			
Complete omission	IE does not occur at all	Patient and/or family not informed about consultation before it occurs	“Sometimes the patients are shocked [when we arrive]. ‘I didn’t know I had heart failure.’ It’s kind of uncomfortable to be the first person to mention it. … It’s easier for the patient to hear that from their [primary] team that they trust and that they’ve been seeing. [When they don’t,] it makes the patient feel … like, ‘Why didn’t the primary team tell me this?’” (Specialist 12)
	Commonly affected IEs: 2, 5, 6, 7, 10, 11		
Stakeholder left out	IE excludes one or more key individuals	Multiple specialist teams consulting on same patient confer with one another about next steps for care but do not loop in primary team	“There will be a conversation between hepatobiliary surgery and advanced endoscopy in GI [gastroenterology], for example. They'll have a whole conversation about … what's going on with the patient's biliary tree and exactly how they're going to approach it. What's going to be done inpatient and what's going to be done outpatient, and who's going to order what … There's a whole plan that's out there in the ether. It's nowhere in the formal medical records … Not only do they [the primary team] not know about this conversation, but they can't help enact any of it, and they can't communicate with the patient [about it].” (Hospitalist 1)
	Commonly affected IEs: 2, 4, 5, 6, 8, 10		
Poorly timed relative to some anchoring event	IE occurs too early or too late relative to other steps of the consultation process, thus reducing the relevance and/or utility of the IE	Primary team requests consultation “too early” in hospitalization	“Sometimes we'll get a consult the day the patient arrives. We'll say, ‘We know you want us to work up this cough and shortness of breath, but they don't even have a CAT scan yet. They don't have labs yet. We're happy to see the patient, but we don't have any information yet. It would be helpful if you had waited just a little bit so that we had something to work off of.’” (Specialist 10)
	Commonly affected IEs: 3, 4, 6, 7, 8, 9		
Content			
Incomplete information	IE lacks information needed by the recipient	A trainee’s preliminary recommendations are not signed off on by an attending	“Often we’ll have some word-of-mouth [i.e., verbal communication from the fellow] … and I don’t know whether their attending is weighing in. Usually they haven’t. Then I’m waiting for the attending to weigh in. The note never shows up, and I don’t know whether the information I’m getting has ever actually even been spoken to from the attending … [Ideally] the attending would weigh in within 24 hours in a documented fashion in the medical charts.” (Hospitalist 5)
	Commonly affected IEs: 3, 9		
Inaccurate information	IE includes inaccurate information	Primary and specialist teams give patient/family conflicting information	“[The primary team] said [to my husband], ‘You’re going to be brought to IR [Interventional Radiology] to have a port put in.’ … [Hours later] a doctor from IR came in and said, ‘I’m from IR. We’re not going to be able to do the port today,’ and left. … Later, my husband’s nurse came in and said, ‘They’re coming to get him from IR [Interventional Radiology].’ And I said, ‘No, they’re not. Some guy [from the IR team] was just in here and said he’s not having it done.’ … It was not a good experience.” (Family 1)
	Commonly affected IEs: 4, 5, 6		
Interpretation			
Misinterpretation	One or more individuals misunderstands the information	Consultation requester misunderstands consultation question	“Oftentimes, the interns [on the primary team] are placing the consult. … They know what they've been told to ask, but they don't have the level of knowledge to get at the subtleties. … The question they ask [the specialist team] is often not the question that their attending wanted.” (Specialist 8)
	Commonly affected IEs: 1, 3, 6, 9, 10		

Abbreviation: IE, information exchange.

In participant accounts, IEs involving patients/families were commonly omitted entirely or carried out in the absence of a key stakeholder, such as a caregiver. Not knowing that the primary team requested a consultation, patients and families found themselves confused by the information coming from different teams, which was sometimes contradictory or heard by them as contradictory (eg, owing to teams using different terminology). Although defects in these IEs made for poor patient/family experiences, they did not present a hard-stop to the consultation. Defects in other IEs, however, were described as more disruptive and sometimes harmful to the consultation’s overall value. For example, some specialists described occasionally being frustrated by consultations that were requested “too late,” such that their input did not stand to make a difference to the patient’s trajectory. According to one specialist, “[A bad consult] comes late. … [The primary team] call and say, ’This patient’s going home today, but we want Oncology to see them.’ You don’t feel that the input you’re giving is important enough to guide the decision on whether or not they even have to stay in the hospital, so why do you need us to see them that day?” (Participant 9).

Similarly, some clinicians reported that, on occasion, defects jeopardize the utility of the consultation. According to one hospitalist, “Sometimes the reason for the consult doesn’t get appropriately relayed or doesn’t get appropriately understood by the consulting [specialist] team. Then they’re not really answering the question you called them for. They’ll focus on some trivial thing, [and you think,] ’That’s great, but you missed the point of the consult.’ It’s very frustrating, and it delays patient care” (Participant 5).

### Theme 3: Contextual Factors

The third theme identified was that contextual factors influence information exchanges, making them more or less prone to defects. Across participants’ consultation experiences, 5 major contextual factors shaped IEs and their susceptibility to defects (Table 3).

**Table 3.** Five Contextual Factors Influencing How Susceptible Information Exchanges Are to Defects

Factor description	Illustrative quotes
Role definition: extent to which interactants have same understanding of the role of primary and specialist team, including each team’s responsibilities and authority	Example 1: Is part of specialist team’s role to help primary team form its consultation question? No: “We often get consults where there really is no definitive question. There’s nothing for us to do. … If we don’t know what you’re looking for, it’s hard for us to provide that.” (Specialist 10) Yes: “The person who's calling [the consult] knows they need help, but they may not know why they need help. They don't know what they’re missing. They don't know what's relevant or not. It's the job of the consultant to help support them through that.” (Hospitalist 4)
	Example 2: Does specialist team have the right to push back against consultations that seem unnecessary and/or ill-suited for the inpatient setting? No: “There are times where I'm like, ‘This is not a negotiation. You might be right that it turns out this consult wasn't needed. But my reasons for needing it may be more than you understand’ … They [specialists] are not supposed to say ‘no’ to any consult.” (Hospitalist 2) Yes: “[Some clinicians] feel that the hospital stay should cover everything. I disagree with that. … So those consults [that say,] ‘Please come assist. History of knee pain.’ It’s like, ‘Really? You're going to bring a trauma surgeon to assist with a history of knee pain for someone who's not injured but [has] had it for six months?’ That's a waste of time and resources and money. That's the kind of patient [for whom] you say [to the primary team, ‘You can call the [outpatient] clinic. … You don't need a[n] [inpatient] consult for that.’” (Specialist 5)
	Example 3: Should the specialist team communicate its recommendations directly to the patient/family? No: “We don’t do that [share our recommendations with the patient and family]. We just write the documentation in the note and hope that the primary team will express it to them.” (Specialist 5) Yes: “I think it's actually preferred for the consultant [specialist] to discuss with the patient what their recommendations are. If they leave it to the primary team to discuss the recommendations, the primary team often can't answer some questions about them.” (Hospitalist 13) Yes, but only using nondefinitive language or after consulting with primary team: “Sometimes consult [specialist] teams come in, see the patient, and then speak in ways that are definitive as if, ‘This is going to happen,’ or ‘That's going to happen.’ … Sometimes it's actually a form of miscommunication, and that sets up expectations that can't be met, or it creates problems that then need to be unworked.” (Hospitalist 12)
Professionalism: extent to which interactants behave in a collegial and/or respectful manner	Example 1: Responding to consultation request “It was just the responsiveness and the respect [that the specialist team showed] for our concerns that was really valuable. They validated our concerns and came to see the patient incredibly quickly.” (Hospitalist 12) “It's annoying when the consultant is either condescending or [acting as if] ‘I don't need to be dealing with this’ kind of thing.” (Specialist 1)
	Example 2: Resolving disagreements about the care plan “[Hospitalists and specialist] are professional people. … Myself and the Infectious Disease doctors sat down, talked it through, and decided, ‘Okay, let's just figure out what's best for the patient.’ I listened to them. They listened to me. We educated each other and tried to come up with some reasonable solution.” (Specialist 2) “The orthopedic surgeon said, ‘Admit to Medicine. Give him some antibiotics, and we're going to tap the hip.’ I said ‘No, you're not going to tap the hip. It's not infected.’ They said to me, ‘But we told the patient we're going to tap the hip.’ … So I said, ‘If you're going to tap the hip, I think this is malpractice, and you can do it on your service, not on mine. I don't want my name associated with this patient.’” (Hospitalist 13)
	Example 3: Communicating with the patient and family “My approach is to say [to the patient and family], ‘These are things that we would be concerned about and would consider for more of an evaluation. … However, we'll have to talk about it with your primary team and see if that's appropriate for your care.’ So some sort of caveat that ‘We're not making the decision. These are things that we're considering … but ultimately, it's up to the primary team.’” (Specialist 3) “The consult [specialist] team or the primary team may … speak about the other team in ways that undermine them. … Something like [the] Infectious Disease [team] saying [to the patient,] ‘We think your primary team is being a little bit cavalier and a little bit aggressive by wanting to put you on IV antibiotics.’ And maybe someone on my team saying, ‘We think the Infectious Disease team is just waffling and they're not able to make up their minds.’ Those sort of snide, underhanded comments that show a disagreement are not good for the patient.” (Hospitalist 12)
Team hierarchy: extent to which trainees are involved in consult	Potential impact on information accuracy, completeness, and timeliness “I think a critical part [of the consult] is that the attending physician [of the specialist team] needs to see the patient to make an evaluation based on a face-to-face evaluation and their own exam and assessment. … Unfortunately, it is not uncommon that people will have a representative [i.e., a trainee] of the [specialist] team go out and make a consulting recommendation, and the [specialist] attending is not there in a timely way. Sometimes, frankly, the advice completely changes once the [specialist] attending sees the patient.” (Specialist 17) “The intern [of the specialist team] is the one who is asked to deliver the recommendations and isn’t always able to explain the reason behind them in a way that the consulting team will understand, and sometimes can’t address [the primary team’s] follow-up questions.” (Hospitalist 6)
Availability of interactants: extent to which interactants are available to engage in a given IE	Example 1: Specialist team availability to conduct consult “Fellows [on the specialist team] often have [outpatient] clinic [duties] on the days that they're doing [inpatient] consults. They'll tell me, ‘I'll come see the patient after I'm done with the clinic.’ So if I call them at 11:00 or 12:00, and they have an afternoon clinic, then they don't see the patient until 5:00 or 6:00, and they tend not to staff the consult until the next day. So there's a real time delay.” (Hospitalist 13)
	Example 2: (Non-) overlap of specialist team and family member availability “I’d try to get in [to be present] for the specialist, but you could never pin them down. It wasn't like they had [an] appointment. I would get in here, and he [my husband] would say, ‘The liver specialist was just here half an hour ago.’” (Family 1)
	Example 3: (Non-) overlap of primary and specialist teams’ availability “Sometimes you get an answer [from the specialist team], and you don't understand why you get the answer that you get. … You’re left with some remaining questions. But it's not always easy or, frankly, convenient to pin down the consultants in a timely manner to get those questions answered.” (Hospitalist 6)
Operational know-how: extent to which interactants know how to move consultation process forward	Example 1: Knowing whom to call “[To consult] some of the surgical subspecialties, you'll end up literally going through 4 or 5 different people to actually find out who's even covering the pager. … It can be really challenging.” (Hospitalist 2)
	Example 2: Knowing what information the specialist team needs up front “Ideally, you provide the consultant [specialist] with the information that the consultant is going to need to be able to answer your question. That's tough because individual physicians on the primary team have varying degrees of background and knowledge … If they [the specialist] know right off the bat that they're going to need … like lab tests or whatnot in order to answer your question, it would be nice if they got that to you quickly.” (Hospitalist 6)

Abbreviation: IE, information exchange.

#### Roles and Boundaries

Although clinicians generally had a shared understanding of which team was responsible for which consultation tasks (eg, all agreed it was the primary team’s decision whether to implement the recommendations), for some duties, participants described variation in teams’ understanding of their respective roles. For example, whereas some participants viewed forming a “good” consultation question as the exclusive responsibility of the primary team, others insisted that the specialist team had an obligation to help with this task, as needed. Tensions rose when specialist teams pushed back against consultation questions they perceived to be overly vague, not pertinent to their specialty, or inappropriate for the inpatient setting. Similarly, variation in each team’s understanding of what, when, and how much information the specialist team should share with the patient/family often led to them receiving mixed messages. Other contested responsibilities included whose job it was to gather needed information (eg, outside hospital records) and implement certain recommendations (eg, scheduling outpatient appointments).

#### Professionalism

The degree to which interactants behaved “professionally” also shaped how IEs unfolded. Some hospitalists described avoiding consulting particular specialties whenever possible because of their reputation for being “rude” (Specialist 1; Hospitalists 7 and 13) or “dismissive” (Specialist 1; Hospitalists 1 and 13), such as making a primary team “feel stupid” (Specialists 2 and 6; Hospitalists 7 and 10). Professionalism—or lack thereof—also surfaced when teams differed in opinion about next steps for care. When one or both teams were unwilling to consider the other’s point of view, participants characterized the disagreement as a “conflict.” However, when each team listened to and acknowledged the other party’s concerns, participants characterized the disagreement as an important part of the consultation process. According to one specialist, “I got consulted recently by Infectious Disease. … They said, ‘The patient needs surgery.’ My contention was, ‘He has a horrible infection in his pelvis for a whole lot of reasons, and surgery will not change any of this. We need to get at the underlying reasons.’ ... [In these situations] we talk it through and decide, ‘Okay, let's just figure out what's best for the patient.’ I listen to them. They listen to me. We educate each other and try to come up with some reasonable solution” (Participant 2).

Professionalism also figured prominently in the experiences of patients and families, who tended to view consultations positively when specialists took the time to introduce themselves, explain their thinking, and answer questions.

#### Team Hierarchy

Clinicians noted that in academic medical centers, consultation work is often divided along team hierarchies, with the least experienced members frequently tasked with communicating the consultation request or recommendations. These participants insisted that, when attending oversight is insufficient, the risk of IE defects (especially inaccurate or incomplete information) increases but that establishing norms and systems to ensure proper oversight could reduce this risk.

#### Availability

Participants from all groups reported that misalignment of stakeholder availability often led to IEs being entirely omitted, excluding key individuals, and/or not occurring at the right time. Specialist teams’ availability was described as particularly difficult to predict, as it depended not only on time of day and day of week (with fewer specialists on service at night and on weekends) but also on whether the specialist team simultaneously attended to patients in its outpatient clinic. Not knowing, for example, when the specialist would arrive to examine the patient often led to both primary team and family members missing the evaluation and not sharing potentially relevant information with the specialist, such as changes in the patient’s status since the consultation request.

### Operational Know-How

Some participants attributed IE defects to insufficient working knowledge or know-how for operationalizing certain parts of the consultation. For example, hospitalists reported that they often do not know what information (eg, test results) specialist teams need up front to begin working on particular consultation questions, leading to incomplete consultant requests and delays. Similarly, both hospitalists and specialists reported occasional delays in IEs owing to not knowing whom to contact on the other team. According to one specialist, “Sometimes even just trying to figure out who’s the ultimate person in charge is confusing. It’s like, ‘What pager number do I need to get a hold of the Medicine attending? Which attending? Which team?’” (Participant 2).

## Discussion

In this qualitative study, participants envisioned the ideal inpatient consultation as a series of perfect information exchanges; however, participants’ actual consultation experiences suggest that this ideal is rarely achieved because IEs are frequently derailed by defects, such as poor timing, incomplete information, and misinterpretation. The ultimate outcome of these defects ranged widely, from temporary confusion, frustration, and minor delays to major issues that jeopardized care quality. Contextual factors influenced smooth progression of the consultation, including the degree to which teams had a shared understanding of each party’s role in the consultation and whether interactants had sufficient working knowledge of the consultation process.

Our findings on contextual factors align with prior work on barriers and facilitators to consultation communication. Salerno et al^13^ found that role assignment was often uncertain and variable, and Miloslavsky et al^17^ found that whether fellows considered teaching part of their role affected their interactions with residents. Professionalism also features prominently in this literature,^11,14,15,16,17^ with several studies identifying pushback from specialists as a major challenge,^11,15,17^ although pushback decreased with increasing familiarity and trust between teams.^11,15,16,17^ Our study adds nuance to the concept of pushback by showing its association with how teams understand their respective roles in the consultation, whereby some specialists viewed questioning seemingly unnecessary consultation requests as part of their responsibility. Although other studies also support our finding that team hierarchy influences whether information is reliably communicated,^14,17^ we also found that the tone of the communication matters, as primary teams were more likely to perceive pushback when specialists expressed reservations about the consultation in a condescending or dismissive manner, a finding that appeared to be independent of rank within teams. We found that negative interactions—such as specialists making hospitalists “feel stupid” for asking questions—seemed to unveil an informal but endorsed hierarchy that exists beyond the parameters of the formal hierarchy of academic medicine and values specialists over generalists (and, in some cases, certain specialists over others). This finding aligns with other studies that have identified “prestige hierarchies” among the medical specialties.^24,25^ Finally, our study identified variation in consultation process both within and across specialties (eg, what information is needed up front). This lack of generalizability in the consultation initiation process may increase the likelihood of false starts and contribute to other defects down the line.

By mapping out information exchange within the ideal consultation, we found that the inpatient consultation process is inherently complicated and potentially prone to failure not only due to the large volume of information and number of people involved, but also the time-sensitive, asynchronous, and multistep nature of the work. Even if each IE were 98% reliable, the overall reliability of the 11-step IE process would be 0.98^11^ or 80%—a failure rate of 20% (assuming independence of failure rates between each IE). If each IE were 95% reliable, nearly 1 out of every 2 consultations would fail to achieve perfect information exchange. In short, the likelihood of 1 or more defects remains high even when every IE is quite reliable. Hospitals seeking to improve consultation efficiency may consider conducting a failure mode and effects analysis to identify where and how the process is failing and assess the relative impact of identified defects.^26^ Improvement interventions should be selected based on the specific defect(s). For example, several studies promote increasing direct communication between teams^11,12,17^; however, this intervention would be ill-suited to address deficits in operational know-how and would potentially exceed what is necessary for a functional IE. That is, the incremental value added by requiring direct communication may not be worth the time cost, particularly if it delays the consultation process. As in the outpatient setting,^27^ indirect and/or asynchronous communication may be sufficient for certain IEs in particular inpatient contexts, such as having the primary team submit consultation requests via an electronic ordering system. Other defects may be identified or addressed via a well-designed electronic medical record that could, for example, automatically flag when an attending specialist has not vetted a trainee’s preliminary recommendations within a specified amount of time. Other defects will likely require interventions at the individual and interpersonal levels, such as training to improve clinicians’ consultation communication skills.^28^

Similar to some quantitative investigations,^1,3,4,8,10^ specialists, patients, and families in our study suggested some consultations did not add value to patient care, although nearly all hospitalists maintained there remained some way to glean value from every consultation. Given the potential harm of unnecessary procedures and the high cost of consultations, eliminating low-value consultations is in the interest of patients, families, and payers. Our study suggests that at least some portion of specialist pushback against consultations may currently serve this gatekeeping function; however, more research is needed to develop acceptable, objective, and reliable gatekeeping mechanisms that ensure rational use but do not block primary teams from obtaining specialist input when they need it.

### Limitations

This study had some limitations. The findings of this single-center study may have limited generalizability to other medical centers, especially nonacademic settings that are resourced differently and may have a different process for (and culture around) conducting inpatient consultations. Participant accounts are subject to recall bias; to mitigate this risk, we restricted eligibility timeframe to 4 months for clinicians and 15 months for patients/family members. We did not collect information on participants' race or ethnicity. Our small, all-female, all-English-speaking sample of Patient and Family Advisory Council members may not have captured the full range of consultation experiences and limits insights into how language barriers affect information exchanges. Future research that includes a larger, more diverse sample of patients and family members—as well as other stakeholders, such as nurses—is needed.

## Conclusions

Successful inpatient consultation requires a complicated, sequenced series of time-sensitive information exchanges that are highly vulnerable to failure. Maximizing the benefit of inpatient consultations will likely entail not only eliminating low-value consultations but also preventing defects that commonly derail the consultation process and lower the value of consultations both to clinicians and to the patients and families they serve.

## Notes

Supplement. **eTable 1.** Results of Second Cycle Coding: Organizing 44 Process-Oriented Codes Into 11 Discrete Information Exchanges **eTable 2.** Consolidated Criteria for Reporting Qualitative Studies (COREQ) Checklist **eAppendix 1.** Interview Guides **eAppendix 2.** Detailed Description of the 11 Key Information Exchanges of an Ideal Consult, Organized by Consult Stage
