# Supplementary material for: Patient and Clinician Perceptions of Factors Relevant to Ideal Specialty Consultations
Source: JAMA Netw Open. 2022 Apr 25;5(4):e228867. doi: 10.1001/jamanetworkopen.2022.8867 (PMC9039767; doi:10.1001/jamanetworkopen.2022.8867)
Supplement: Supplement. — eTable 1. Results of Second Cycle Coding: Organizing 44 Process-Oriented Codes Into 11 Discrete Information Exchanges eTable 2. Consolidated Criteria for Reporting Qualitative Studies (COREQ) Checklist eAppendix 1. Interview Guides eAppendix 2. Detailed Description of the 11 Key Information Exchanges of an Ideal Consult, Organized by Consult Stage [file jamanetwopen-e228867-s001.pdf]

Supplemental Online Content

Roche SD, Johansson AC, Giannakoulis J, et al. Patient and clinician perceptions of factors relevant to ideal specialty consultations. *JAMA Netw Open.* 2022;5(4):e228867. doi:10.1001/jamanetworkopen.2022.8867

eTable 1. Results of Second Cycle Coding: Organizing 44 Process-Oriented Codes Into 11 Discrete Information Exchanges

eTable 2. Consolidated Criteria for Reporting Qualitative Studies (COREQ) Checklist

eAppendix 1. Interview Guides

eAppendix 2. Detailed Description of the 11 Key Information Exchanges of an Ideal Consult, Organized by Consult Stage

This supplemental material has been provided by the authors to give readers additional information about their work.

© 2022 Roche SD et al. *JAMA Network Open.*

eTable 1. Results of second cycle coding: Organizing 44 process-oriented codes into 11 discrete information exchanges

Consult Stage	Process Code		Information Exchange(s)
Identification of Consult Need & Consult Preparation	1.	Is there a clearly defined consult question?	1
	2.	Is the timing of the proposed consult appropriate given the patient's trajectory and consult turnover time?	1
	3.	Has the patient and/or family been appropriately involved and educated about the proposed consult (e.g., what a consult is, what the process is like)?	2
	4.	Does the patient/family agree with obtaining the consult?	3
Consult Request	5.	Is there a clear process for requesting a consult?<br>If so, is the consult request process standardized across all specialties? Is it well-understood by all users? Who can initiate this process?	3
	6.	If in a training setting, does the consult request process have appropriate supervision built into it (e.g., to ensure trainee conveyed the consult question as intended)?	3
	7.	Is the process for obtaining a consult responsive to urgency?	5, 7
	8.	Does the request contain all of the information the specialist team needs (e.g., consult question, patient MRN, level of urgency)?	3
	9.	Is the consult request accepted by the specialist team?	3
	10.	Is there a shared understanding about what the consult question is?	3
	11.	If in a training setting, is the consult request reviewed by a specialist team member with sufficient experience to confirm that their service can address the question asked?	4
	12.	Is there agreement between the primary and specialist team on the need for specialist evaluation in the inpatient setting?	3
	13.	Has the specialist team assessed available data and identified gaps in information needed to carry out the consult (e.g., tests needed)?	3
	14.	Does the specialist team indicate to the primary team an expected timeframe for the consult occur? If so, is this timeframe reliable?	3
	15.	Have both teams considered the potential outcomes of the consult?	3
	16.	Is an expected timeframe for the consult to occur communicated to the patient/family?	5
	17.	Is there documentation of (1) the consult request and (2) the specialist team's receipt of it?	3
Patient Evaluation and Data Collection	18.	Does the specialist arrive in a timely manner given the consult's urgency?	6

© 2022 Roche SD et al. *JAMA Network Open.*

	19.	Is the consult adequately timed such that the patient is available when the specialist arrives and family members are present, if desired?	6
	20.	Is the primary team available to answer the specialist team's questions, if needed?	7
	21.	Has the specialist team indicated whether they are going to continue to follow the patient?	7
	22.	Is there documentation indicating to the primary team that the specialist has seen the patient?	7
	23.	Does the specialist arrive with sufficient knowledge of the case prior to beginning his/her evaluation?	7
Recommendation Formation, Consensus-Building, and Finalization	24.	Are the recommendations formulated and documented in a timely manner given the consult's urgency?	9
	25.	If multiple specialist teams are involved in a single patient's care, is there a shared understanding about how the teams will collaborate and who will be responsible for organizing/facilitating cross-service discussion?	8
	26.	Is the primary team available to discuss with the specialist team the proposed recommendations?	9
	27.	Does the specialist team clearly communicate its recommendations to the primary team, including the rationale for its recommendations?	9
	28.	Is the specialist team available to answer any follow-up questions the primary team has?	9
	29.	Do the primary and specialist teams reach a consensus and shared understanding about next steps for care?	9
	30.	Are there shared expectations between the primary and specialist teams about how the recommendations will be communicated to the patient/family?	9
	31.	Are the specialist team's recommendations shared with the patient/family?	10
	32.	Is the information received by the patient/family consistent across care teams?	10
	33.	Is there opportunity for the patient and/or family to ask questions about the proposed next steps for care?	10
	34.	If necessary, are recommendations modified based on patient/family feedback?	10
	35.	Are the recommendations consistently documented in the same place in the medical record?	9
	36.	Are the recommendations entered into the medical record in a timely manner?	9
	37.	If consult requires supervision, is there documentation of the supervisor's oversight and approval of the evaluation and recommendations?	9
	38.	Do the specialist team's recommendations address the consult question asked?	9
	39.	Are the recommendations specific, decisive, and thorough?	9
	40.	Are the recommendations contextualized and patient-/family-centered?	9

© 2022 Roche SD et al. *JAMA Network Open.*

Recommendation Implementation	41.	If the specialist team's recommendations are not implemented by the primary team, is the reason for non-implementation shared with the specialist team?	11
	42.	If the specialist team's recommendations are not implemented by the primary team, is the reason for non-implementation shared with the patient/family?	11
	43.	Is there clear documentation indicating when the consult has ended?	9
	44.	When needed, does the specialist team assist with implementing recommendations (e.g., scheduling outpatient care)?	9

eTable 2. Consolidated criteria for reporting qualitative studies (COREQ) checklist

Domain	No. Item	Study Details	Section Where Reported
Research Team and Reflexivity	***Personal Characteristics***		
	1. Interviewer/facilitator	A trained research assistant (SR) conducted all interviews.	Methods
	2. Credentials	SDR has a Master's in Public Health and a BA in Cultural Anthropology. She is currently a PhD candidate in Global Health Implementation Science. ACJ and JSP were both involved in data analysis and interpretation. ACJ has a PhD in Sociology. JPS is a medical doctor and board-certified pulmonologist.	Title Page
	3. Occupation	SDR: Research analyst and PhD candidate (University of Washington); ACJ: Instructor in Medicine (Harvard Medical School) and Director of Social Science Research (Beth Israel Deaconess Medical Center); JPS: Assistant Professor of Medicine (Harvard Medical School) and Director of the Center for Healthcare Delivery Science (Beth Israel Deaconess Medical Center)	Not included
	4. Gender	Female	Methods
	5. Experience and training	SDR, ACJ, and JPS have graduate-level training in qualitative research and prior experience collecting, analyzing, and publishing results of qualitative research studies.	Methods

	Relationship with Participants		
	6. Relationship established	Relationships were limited to interviews.	Not included
	7. Participant knowledge of interviewer	Prior to interviews, SDR introduced herself as a research assistant from the medical center's Department of Health Care Quality. No other personal details of the interviewer were shared with participants.	Not included
	8. Interviewer characteristics	No interviewer characteristics are reported.	Not included
Study Design	***Theoretical Framework***		
	9. Methodological orientation and theory	Our approach is informed by Corbin and Strauss's (2015) content analysis and Hsieh and Shannon's refinement of it. In line with this approach, we collected data primarily through open-ended questions and derived our coding categories directly from the text data.	Methods
	Participant Selection		
	10. Sampling	We purposively sampled hospitalists and specialists who had requested or performed, respectively, an inpatient consult during the previous four months. We also used snowball sampling, asking physician participants at the conclusion of the interview for suggestions as to whom else we might consider interviewing. We purposively sampled patients who had been admitted to the medical center during the previous 15 months and received a specialist consult and family members of patients meeting the aforementioned criteria.	Methods
	11. Method of approach	For physician participants: A research assistant (RA) sent an e-mail describing the study objectives, eligibility criteria, and details of participation to the medical center's hospitalist and medical residency program listservs. This RA then e-mailed individuals who expressed interest in participating to confirm eligibility and schedule an interview. For patient and family participants: We had the medical center's Director of Patient and Family Engagement send an e-mail to the medical center's	Methods

© 2022 Roche SD et al. *JAMA Network Open.*

		Patient-Family Advisory Council (PFAC) listserv describing the study objectives, eligibility criteria, and details of participation. The RA then e-mailed individuals who expressed interest in participating to confirm eligibility and schedule an interview.	
	12. Sample size	38 total interviews completed	Results
	13. Non-participation	Two hospitalists, two specialists, and one patient expressed interest in participating but were unable to due to scheduling conflicts.	Not included
	Setting		
	14. Setting of data collection	All interviews took place either in a private conference room at the medical center or via telephone call.	Methods
	15. Presence of non-participants	Only the participant and interviewer were present during the interviews.	Not included
	16. Description of sample	17 specialists, 13 hospitalists, 4 patients, and 4 family members. Details about participant sex, field of specialization (for specialists), and relationship to patient (for family members) are included in the Results section.	Results
	Data Collection		
	17. Interview guide	We developed and pilot tested a de novo interview guide for each participant group.	Methods
		Pilot test interviews were not included in the final dataset, and individuals who participated in a pilot test interview were not subsequently invited to participate in an interview for the study.	Not included
	18. Repeat interviews	Each participant was interviewed once (i.e., no repeat interviews).	Methods
	19. Audio/visual recording	We audio-recorded all interviews with participant consent.	Methods
	20. Field notes	The RA took notes during the interview in case the recording equipment failed, but no such failures occurred, and the notes were not used in the analysis.	Not included
	21. Duration	Median interview duration was 29 minutes (IQR: 24-34 minutes).	Results
	22. Data saturation	We did not conduct interviews to the point of data saturation.	Not included
	23. Transcripts returned	We did not return transcripts to participants for comment or correction.	Not included
	Data Analysis		
	24. Number of data coders	Two: SDR and ACJ.	Methods

© 2022 Roche SD et al. *JAMA Network Open.*

Analysis and Findings	25. Description of the coding tree	Our final codebook included 44 codes—phrased as yes/no questions—that captured information exchange processes and five codes that captured socio-behavioral factors that influence the consult process. In the online supplement, **eTable 1** shows the results of our second-cycle coding, through which we organized the 44 process codes into 11 discrete information exchanges.	Methods and eTable 1 in the online supplement
	26. Derivation of themes	Our reported themes emerged directly from the text data.	Methods and Results
	27. Software	NVivo 12 (QSR International, Burlington, MA)	Methods
	28. Participant checking	We did not provide participants with feedback on our findings.	Not included
	Reporting		
	29. Quotations presented	We present participant quotes illustrating our findings. Each quote is accompanied by a participant number.	Tables 1 and 2
	30. Data and findings consistent	Yes	Results
	31. Clarity of major themes	Our three major themes are identified by subheadings within the Results section. We discuss the relevance of these themes in the Discussion section.	Results and Discussion
	32. Clarity of minor themes	We do not discuss minor themes in this paper.	Not included

© 2022 Roche SD et al. *JAMA Network Open.*

Appendix A: Interview guides

Family Interview Guide

1. Around when were you/your family member hospitalized here at BIDMC? (We don't need exact dates. Just an estimate is fine.)
2. Do you recall what floor you were/your family member was on?
3. During your/their hospitalization, were any specialists involved in their care?
   a. What kind of specialist(s)?
      (If interviewee mentions more than one specialist, then say, "Was there one specialist that you remember the most, for whatever reason?" *If yes, then say,* "Ok great. For the rest of my questions, could you focus on just the care that you/your family member received from this specialist team?")
   b. When did you first learn that a [specialist] was going to be involved in your/your family member's care? (*Probe if needed: Were you informed by a physician member of the primary team, or a nurse?)*
   c. *(If not answered above:)* Were you told about the possibility of specialist involvement ahead of time?
   d. Did you understand why the specialist was being called in?
   e. Did you think that involving a specialist was a good idea?
   f. About how much time passed between when you learned that the specialist was going to be involved and when s/he arrived? Were you satisfied with this timing?
   g. Once the specialist arrived, can you describe what happened? Did s/he share their thoughts about your/your family member's care?
   h. *(If applicable)* After the specialist came, did you find that the information you received from the specialist matched the information you received from the primary team?
4. As far as you know, was there any disagreement between the specialist and the primary team regarding your/your family member's care?
   If yes:
   a. How did you know there was disagreement?
   b. Do you think this disagreement impacted the quality of the care you/your family member received?
5. After the specialist was involved, did anything change about your/your family member's care? For example, did the primary team conduct additional tests or make medication adjustments?
   If yes:
   a. Do you think these changes were good for you/your family member's care? Why/why not?
6. Overall, do you think it was worthwhile to involve the specialist? *(If participant doesn't understand question, ask "Do you think involving a specialist helped you/your family member?")*
   If yes:
      a. What do you think was gained by involving a specialist?
   If no:

© 2022 Roche SD et al. *JAMA Network Open.*

   b. What, if anything, could have been done to make the specialist's involvement worthwhile?
7. Do you think the specialist care went well?
   If yes:
   a. What about the consult made you feel it was high quality?
   If no:
   b. What went poorly?
8. What, if anything, would you have changed about the specialist care you received? *(If participant says "nothing" then ask: "Do you feel like everything about the consult was the best you could possibly imagine?")*
   Probes:
   - Would you have changed…
     - who was involved?
     - what information was exchanged?
     - the timing?
9. Sometimes there is a delay between when the specialist sees the patient, and when the specialist is able to make a recommendation about the patient's care. For example, sometimes the specialist needs additional information from tests before s/he can make a recommendation for care. In this situation, what is the best way to close this communication loop? For example, would you prefer the specialist to return to explain his or her findings? Would you prefer to hear about the findings from the head doctor? From the nurse?

10. As you know, sometimes one [specialist] may see the patient on one day, but then a different [specialist] may follow up with the patient on the next day. As a patient/family member, how do you feel about this transition of doctors?
11. Is there anything else you'd like to share about your experience of specialist care?

Physician Interview Guide

1. From your perspective as a clinician, what would the perfect consult look like?
   Probes:
   - *Who is involved?*
   - *What information would each person have?*
   - *How does the consult affect the patient's care?*
   - *Among the things that a perfect consult comprises, is being "worthwhile" one of the things?*
2. Thinking back to the last time you were on service, did any consults go like this?
   If yes:
   - *Can you walk me through how that consult went?*
3. In what ways might a consult go poorly for the team?
   Probes:
   - *What are some things that could go poorly during the request? The delivery? The opining?*

© 2022 Roche SD et al. *JAMA Network Open.*

- *Does the point in which the consult occurs during the patient's hospitalization matter (e.g., consults on day of admission, day of discharge)?*
- *In general, are consults impacted by shift change?*
- (For primary team physicians only:) *Do you see any difference in consults that are conducted by a house staff team versus a hospitalist-only team?*

4. Is there anything we could change about how we do consults to avoid some of the issues you just described?
5. Now I want you to imagine that you are a patient or family member. What would a perfect consult look like from that perspective?
   Probes:
   - *With whom do they interact?*
   - *What information do they receive? From whom?*
   - *What is the outcome of the perfect consult?*
6. In what ways might a consult go poorly for the patient or family?
   a. What factors do you think contribute to [X] happening?
      (e.g., if they say "mixed messages", ask: "What factors contribute to patients and families receiving mixed messages?"
   b. Is there anything we could change about how we do consults to avoid this from happening?
7. (For primary team physicians only:) Do you think about different consulting services differently (for example, surgery versus medicine, or one division within a department versus another division)?
   Probes:
   - *Are there some services where you only want to talk to a specific person and others where you can talk to anyone?*

   a. (If yes:) Why do you think about them differently?
   b. (If applicable:) How does the culture of a consulting service affect their consults?
8. In your experience, are there some consults in which the costs outweigh the benefits?
   Probes:
   - *What are the costs in this scenario?*
   - *What are the benefits?*

   a) Have you ever ordered/performed consults that you did not think were needed? If so, why?
   b) After a consult was obtained/performed, have you ever thought they were unhelpful or did not add to the patient care? If so, please describe the consult and why you didn't think it wasn't helpful.
9. Think back to a consult in which somebody had a conflict with someone else, for example, a conflict between the consulting team and the primary team, or between consultants. Could you tell me about that conflict and what effect you think it had overall?
   Probes:
   - *Who was in disagreement? About what?*
   - *How was the disagreement resolved?*

© 2022 Roche SD et al. *JAMA Network Open.*

- *What effect, if any, do you think the conflict had on patient care?*
- *Do you think the patient/family were aware of the conflict?*

Appendix B: Detailed description of the 11 key information exchanges of an ideal consult, organized by consult stage

Stage 1: Identification of Consult Need

Physicians explained that, in the ideal consult, the primary team identifies the need for specialist input and forms a consult question that is “specific”, “clear”, and “well-defined.” Specialists, in particular, stressed that the ideal consult would be for a problem that the primary team cannot resolve on its own and for which specialist input has the potential to impact the course of the patient’s inpatient hospitalization. In IE 1, the primary team discusses the need for a consult amongst themselves and tasks a member—in academic medical centers, often a trainee—with inputting the consult request. In IE 2, the primary team explains to the patient/family why they want specialist input and what the consult will entail. The patient/family, in turn, confirms that they understand why the consult is being sought and shares any objections to or questions about the consult.

Stage 2: Consult Request

In IE 3, the primary team contacts the specialist team member responsible for fielding consult requests—often a trainee in academic medical centers. The primary team member indicates who the patient is, what the consult question is, and any other information relevant to the consult (e.g., test results). The specialist team member confirms that he or she understands the consult question, indicates whether the specialist team needs any other information, and provides an expected timeframe for when the specialist team will evaluate the patient. In some consults—such as ones in which the person fielding the consult request is not the person who will be conducting the consult—IE 4 occurs, with the specialist team member relaying the

© 2022 Roche SD et al. *JAMA Network Open.*

consult question to other members of the specialist team, and the team developing a plan for conducting the consult. In IE 5, the primary team communicates the expected timeframe for the consult to the patient/family with the caveat that it is subject to change.

Stage 3: Patient Evaluation

In IE 6, the specialist arrives to evaluate the patient and solicit information from the patient/family, as needed. The patient/family answers the specialist's questions and asks the specialist any questions they have. In IE 7, the specialist team gathers any further information needed from the primary team and lets the primary team know when they can expect to receive final recommendations.

Stage 4: Recommendation Formation, Consensus-Building, and Finalization

IE 8 occurs when trainees conduct the initial patient evaluation and/or when multiple specialist teams are consulting on the patient. In the former situation, the trainee shares his or her preliminary recommendations with the specialist attending who either agrees with or modifies the recommendations. In the latter situation, each specialist team shares its preliminary recommendations with the other, and the teams come to a consensus about what recommendations to make to the primary team. In IE 9, the specialist team communicates its final recommendations to the primary team and answers any clarifying questions.

Stage 5: Recommendation Implementation

Lastly, in IE 10, the primary team and/or specialist team communicates the updated care plan to the patient/family. The patient/family indicates whether they agree with the plan and asks any questions they have. Specialists reported that the ideal consult would also include IE 11, in which the primary team, if it decides against following the specialist team's recommendations, explains to the specialist team why they did not implement the recommendations.

© 2022 Roche SD et al. *JAMA Network Open.*
